# Prevalence of Hepatitis C Virus Infection in a Surgical Population of Southeast China: A Large-Scale Multicenter Study

**DOI:** 10.1155/2020/8219536

**Published:** 2020-04-15

**Authors:** Ping Chen, Yang Zheng, Yubo Cai, Pengfei Zou, Nan Li, Conggao Peng, Hainv Gao, Jimin Liu, Yongping Chen, Zhaowei Tong, Lanjuan Li

**Affiliations:** ^1^Shulan Hospital, Hangzhou 310012, China; ^2^The Third People's Hospital of Zhengzhou, Zhengzhou 450000, China; ^3^Jiulongpo People's Hospital, Chongqing 400051, China; ^4^Zhejiang University School of Medicine, Hangzhou 310058, China; ^5^Department of Pathology and Molecular Medicine, Faculty of Health Sciences, McMaster University, 1280 Main Street West, Hamilton, Ontario, Canada; ^6^Department of Infectious Diseases, The First Affiliated Hospital of Wenzhou Medical University, Hepatology Institute of Wenzhou Medical University, Wenzhou Key Laboratory of Hepatology, Wenzhou 325000, China; ^7^Huzhou Central Hospital, Huzhou 313000, China; ^8^State Key Laboratory for Diagnosis and Treatment of Infectious Diseases, Collaborative Innovation Center for Diagnosis and Treatment of Infectious Diseases, The First Affiliated Hospital, College of Medicine, Zhejiang University, Hangzhou 310003, China

## Abstract

**Background:**

Chronic HCV infection affects 80 million people globally and may progress to advanced liver disease. The present study aims to investigate the present epidemiology of HCV infection in a southeastern Chinese surgical patient cohort.

**Methods:**

Blood samples obtained from 78,484 surgical patients from 18 different city and county hospitals were enrolled. The incidence of serum HCV antibody positivity, HCV RNA load, and HCV genotyping, as well as demographics and relevant clinical history, were investigated. Data were stratified using the multistage cluster random sampling method and further analyzed using the SPSS-20 package.

**Results:**

HCV antibody positivity was detected in 0.15% of the population (95% confidence interval (CI): 0.12%–0.18%). Genotype 1b (55.74%) was the dominant type. The HCV infection peaked in the age groups of 16–20, 41–50, and 61–65 years, and it was higher in males than in females (0.19% *vs.* 0.13%, *P* < 0.05). The geographical distribution of infection rates differed: 0.19% (95% CI: 0.14%–0.24%), 0.18% (95% CI: 0.13%–0.23%), and 0.06% (95% CI: 0.03–0.09%) in plain areas, islands, and valley regions, respectively. Patients with transfusion history and urban residence were associated with high HCV RNA levels (adjusted odds ratio = 11.24 and 6.20, *P* < 0.05).

**Conclusion:**

The prevalence of HCV infection in this cohort from southeast China was 0.17%, which is lower than the reported 0.43% infection rate in China in 2006. This result can be (partially) explained by the improvement of blood donor screening and the successful campaign for the use of disposable syringes and needles.

## 1. Introduction

Chronic hepatitis C is an infectious disease that affects 80 million people globally. Hepatitis C virus (HCV) is mainly transmitted by contacting blood, including transfusion, acupuncture, and intravenous drug use (IVDU) [1]. The HCV transmission route varies in different countries. For example, IVDU is the most common in the United States and West Europe, while iatrogenic spread is the highest in Japan [2–4]. A majority of HCV-infected patients have become chronic, leading to advanced liver diseases, which include 15–35% of patients with cirrhosis, after 25–39 years of infection [5, 6], and 1–7% of them may progress to hepatocellular carcinoma (HCC) [1, 7, 8].

The highest HCV antibody-positive rate in the world was detected in Egypt, in which 18% of people under the age of 18 and >50% of people over the age of 30 were positive [9–11].

Nationwide surveys in 1991 and 2006 revealed that HCV antibodies were detected in 3.20% and 0.43% of the general population, respectively, in mainland China [12]. A cross-sectional study in 2009 revealed that 0.6% were HCV antibody positive, suggesting that China had already jointed the rank of countries with a low HCV infection rate [13].

However, China remains to have the largest number of HCV-infected patients (29.8 million) in the world due to its nearly 1.4 billion population [14].

Different from the hepatitis B virus (HBV) vertical transmission mode in China (i.e., mother-to-children transmission, MTCT), blood transmission represents as the main route of HCV transmission [15–18]. Commercial blood donors used to be the main transmission source in China. However, after implementing the mandatory blood donor screening program in 1998, hemodialysis and the use of intravenous drugs have gradually replaced transfusion as a major HCV transmission source [19–22]. For instance, IVDU-induced HCV infection has continuously increased in southern China since 2012 [23].

Southeast China is one of the most developed regions in the country and features with a high population density and complex geographic landscapes [24]. A nationwide study in 2006 revealed that the HCV infection rate was 0.27% and 0.29% in East and South China, respectively, and both lower than that in other regions [25].

There have been merely few large-scale epidemiologic studies on HCV infection in China, nationally or regionally, since 2006. A meta-analysis published in 2011 reported 0.79% (95% confidence interval [CI]: 0.30–1.51%) of the HCV antibody-positive rate in volunteer blood donors [17].

Hence, it is necessary to systematically analyze the prevalence and genotypes of HCV infection in Southeast China, in order to update the HCV molecular epidemiology in this economy-vibrant region.

The present study investigated the prevalence of HCV infection, HCV RNA load, and viral genotype of 78,484 preoperative patients from 18 city or county hospitals in the Zhejiang province in China. By analyzing these data, the investigators aimed to determine the prevalence of HCV infection among preoperative patients in this region.

## 2. Material and Methods

### 2.1. Study Population

All preoperative patients from 18 city or county hospitals in the Zhejiang province from May to July 2017 were enrolled in the present study.

Patients' demographics (age, gender, occupation, and address) and laboratory results (HBV, HCV, human immunodeficiency virus, and syphilis tests performed before surgery) were initially collected. In this study, patients with positive HCV antibodies were considered as infected people, so we used the anti-HCV positivity to present the infection of HCV, and we also used the third-generation hepatitis C antibody test kit and disposable syringe and needle to test the anti-HCV in serum. For infected patients, their blood samples were further evaluated for HCV RNA level and genotype, as well as other laboratory tests such as albumin, alanine aminotransferase (ALT), aspartate aminotransferase (AST), total bilirubin, white blood cells (WBC), and platelets. Relevant clinical history, such as substance abuse, hemodialysis, transfusion and medications for HCV, and abdominal ultrasound results were also collected.

### 2.2. Sampling Strategy

The multistage cluster random sampling method was utilized for the present hospital-based study.

Three steps were performed to select the population for the investigation: Step I: the selection of participating cities or counties. County-level cities were ranked into three tiers according to economic level, and six cities were randomly selected for each tier. Step II: the selection of participating hospitals in these selected cities. The local central hospital with the largest outpatient population was selected as the survey site in each city. Step III: the selection of surgical patients in these selected hospitals as study subjects. The potential participant size was estimated according to the monthly average number of surgical patients admitted to each hospital in the prior three months and was proportionally distributed. The enrolled patients were selected among the preoperative patients.

Inclusion criteria: (1) informed consent; (2) no restriction on age or gender; (3) living in Zhejiang province for more than six months; (4) admitted between May and July 2017.

Exclusion criteria: (1) subjects who received “preoperative standard tests” (HBV, HCV, HIV, and syphilis) but no surgery was performed due to clinical conditions; (2) patients whose operation time fell within the enrollment period, in which “preop standard tests” were conducted earlier; (3) outpatients who underwent minor operations; (4) nonlocal residents who received surgical treatment.

The sample size was calculated using the following formula:

(1) $$\begin{array}{c} n=\frac{Z_{\alpha /2}^{2}\left(1-p\right)}{\varepsilon^{2}p}\times \text{Deff}=\frac{Z_{\alpha /2}^{2}\left(1-p\right)}{\delta^{2}}\times \text{Deff,} \end{array}$$

where estimated probability: *P*=0.29, permissible error: *δ* = 0.003, confidence level: 1 − *α* = 95%, and designed effect coefficient: Deff = 1.5).

The average HCV infection prevalence in East China from the last nationwide epidemiologic study in 2006 was used as the estimated probability [25]. The power calculation revealed that a minimum of 1,850 patients were needed to reach 95% power.

HCV RNA cutoff level of 10*E* + 05 IU/ml was used to separate HCV positive samples into high and low HCV RNA groups. Either ultrasound or APRI > 2 was used to determine cirrhosis patients.

**Figure 1:**
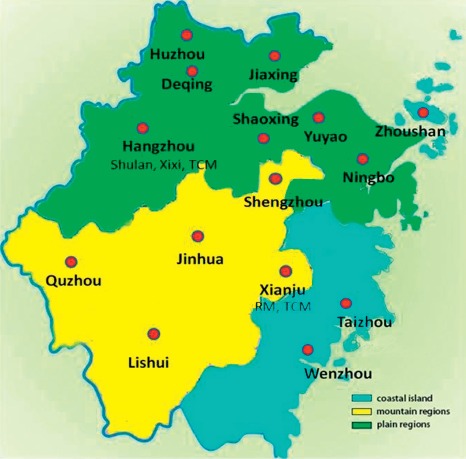
The classification of terrains in the Zhejiang province.

All 18 areas in the present study were categorized into three terrains, namely, plain, hill/mountain, and coastal islands, following the terrain classification data of the Zhejiang Geological Bureau. Among these, Hangzhou, JiaXing, Huzhou, Deqing, Ningbo, Yuyao, and Shaoxing are plain regions, Xianju, Shengzhou, Lishui, Jinhua, and Quzhou are hill/mountain regions, and Wenzhou, Taizhou, and Zhoushan are coastal islands (Figure 1).

### 2.3. Serology Lab Testing

Blood samples were transported in a low-temperature container directly to Tongchuang Clinical Laboratories (Hangzhou, China) from the selected hospitals.

The HCV antibodies were tested using ELISA kits (WANTAI BioPharm Co., Beijing, China), and positive and indeterminate samples were confirmed or excluded by other ELISA kits (Abbott, USA). S/CO ≥ 1.0 (sample/cut-off) was considered as positive.

### 2.4. Molecular Lab Testing

HCV RNA was tested by RT-PCR (Xi'an Tianlong Science and Technology Co., Ltd., Xi'an, China), operation is done strictly in accordance with the instructions, and the lower limit of detection was 50 IU/ml.

The HCV genotypes were determined by PCR-sequencing (Da An Gene Co., Ltd. of Sun Yat-Sen University, Guangzhou, China). This genotyping kit could distinguish all types, as long as the HCV RNA level exceeds 1,000 IU/ml.

### 2.5. Statistical Analysis

All data were entered into a Microsoft Excel spreadsheet and analyzed using IBM SPSS statistics 20. Demographic data and genotypes were analyzed by descriptive statistics. The normality of the distribution was evaluated using *P*-*P* graph, and data with a normal distribution were presented as mean ± standard deviation (*x* ± SD). Differences in HCV infection among the different groups were determined using the chi-square test. The Fisher exact test was used to determine the difference in HCV RNA among groups with different genotypes. Univariate and multivariate analyses were performed to identify the risk factors that determine the HCV RNA level and cirrhosis using a stepwise logistic regression model. *P* < 0.05 was considered statistically significant. All the figures were plotted using GraphPad Prism 7.

## 3. Results

### 3.1. Characteristics of Participants

The total number of samples valid for this investigation was 78,484. Among these, 35,583 were males and 42,901 were females (ratio: 0.83 : 1), and the average age was 48.37 ± 18.80 years old.

HCV antibody positivity was 0.15% in the study population (95% CI: 0.12%–0.18%).

**Figure 2:**
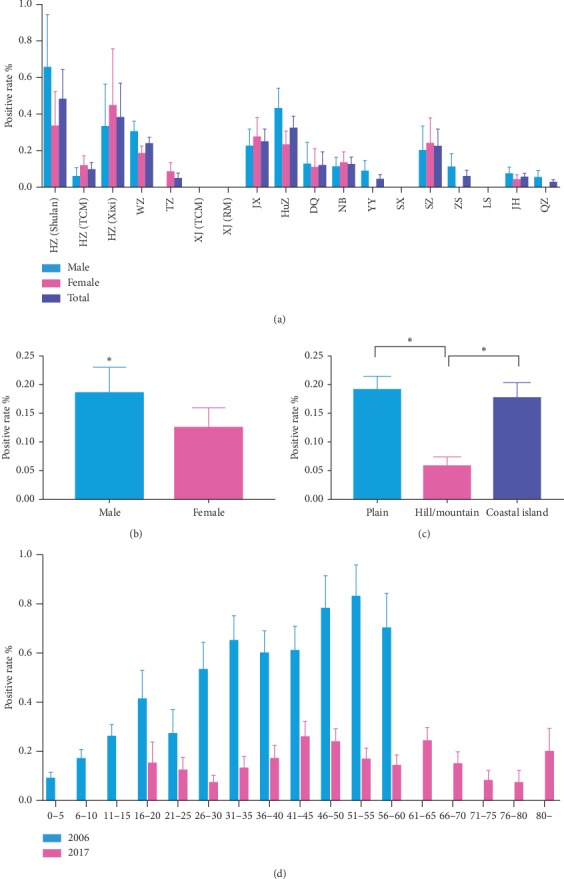
(a) HCV infection rates in the 18 regions: The letters are the abbreviations of the regions. HZ: Hangzhou; WZ: Wenzhou; TZ: Taizhou; XJ: Xianju; JX: Jiaxing; HuZ: Huzhou; DQ: Deqing; NB: Ningbo; YY: Yuyao; SX: Shaoxing; SZ: Shengzhou; ZS: Zhoushan; LS: Lishui; JH: Jinhua; QZ: Quzhou. The letters in brackets represent different hospitals. Shulan: Hangzhou Shulan hospital; TCM: Traditional Chinese Medicine hospital; Xixi: Hangzhou Xixi hospital; RM: Renmin hospital; (b) HCV infection in gender groups; (c) HCV infection among the three different terrains; (d) HCV antibody-positive rates among the different age groups in the present study and in 2006.

The anti-HCV antibody positivity among different geographic regions and genders is illustrated in Figure 2(a). Two hospitals in Hangzhou reported the highest prevalence (0.48% and 0.38%, respectively), while Xianju, Shaoxing, and Lishui reported no positive case. The difference in HCV infection among these different regions was statistically significant (*X*^*2*^ = 70.11, *P* < 0.001).

It was also observed that HCV infection in males (0.19%, 95% CI: 0.14%–0.24%) was significantly higher than that in females (0.13%, 95% CI: 0.10%–0.16%) (*X*^*2*^ = 4.534, *P*=0.033; Figure 2(b)).

The HCV-infected population was 0.19% (95% CI: 0.14%–0.24%) in the plain area, 0.18% (95% CI: 0.13%–0.23%) in the coastal islands, and 0.06% (95% CI: 0.03%–0.09%) in the valley. HCV infection in plains and coastal islands were significantly higher than those in hills/mountains (*X*^*2*^ = 16.72, *P* < 0.001; Figure 2(c)).

Figure 2(d) presents the distribution of participants and positive rates at different age groups. Patients who were 16–20, 41–50, and 61–65 years old had the highest HCV infection, while no HCV antibody was detected in patients under the age of 15. Furthermore, the HCV infection in all age groups in the present study was lower, when compared to the HCV prevalence released by the 2006 nationwide study.

### 3.2. Characterization of HCV Antibody-Positive Samples

**Figure 3:**
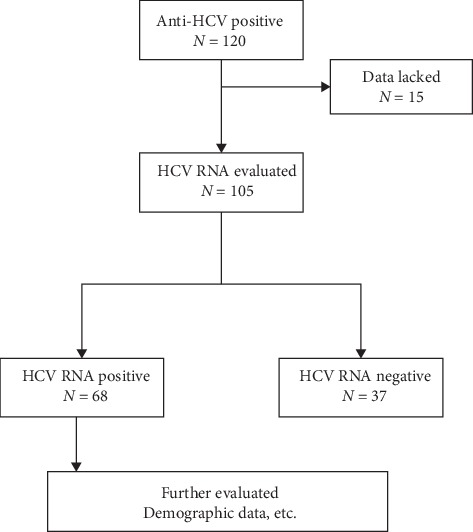
The fluxogram of patients.

A total of 120 subjects were HCV antibody positive. Among them, 15 subjects lacked the data, while 37 subjects were HCV RNA negative, suggesting past infection. The remaining 68 subjects had complete information and were subjected to characterization, including the determination of HCV RNA levels and genotypes, the coinfection with other viruses, infection route, recent clinical resources of CBC, liver function tests, and abdominal images (Table 1 and Figure 3).

There were 16 (23.5%) patients with cirrhosis diagnosed by either ultrasound or APRI > 2. HCV RNA level of 10*E* + 05 IU/ml was used as a cutoff to separate low- and high-level HCV RNA, according to previous studies [8, 26–28]. Then, factors associated with cirrhosis development and the HCV RNA level were assessed.

Genotype 1b (*n* = 34, 55.74%) was the most prevalent in the Zhejiang province, followed by type 6a (*n* = 15, 24.59%). However, the difference in the HCV RNA level among these different genotypes was not statistically significant (Fisher exact test, *P*=0.71).

The univariate analysis indicated that age, gender, transfusion history, and residential area (rural or urban) were independent factors associated with the HCV RNA level. The stepwise logistic regression adjusted by gender and age suggested that the transfusion history (aOR = 11.24, *P*=0.03) and urban area (aOR = 6.20, *P*=0.02) were associated with the high HCV RNA level (Table 2).

Furthermore, none of the demographic variables were identified to be a determinant for developing cirrhosis. Albumin, ALT, and AST were associated with cirrhosis. Given the fact that low albumin level reflects liver cirrhosis, while an elevated AST level may not be liver specific, the elevated ALT level was selected as a risk factor of cirrhosis (aOR = 1.03, 95% CI:1.01–1.05, *P*=0.01) (Table 3).

## 4. Discussion

The percentage of HCV antibody-positive subjects in all age groups in Southeast China was 0.15% in the present survey, which is slightly lower than that in previous studies [25, 29]. The reduced HCV infection fits the trend of the progressive decrease of HCV infection in China over the last decades, which was the result of the strict implementation of voluntary blood donation, and the screening of blood donors with HBV, HCV, and HIV infection. In particular, HCV was screened with the third-generation hepatitis C antibody test kit and use of a disposable syringe and needle [20, 30]. However, a recent study suggested that the annual average incidence of hepatitis C in China in the last 10 years after the SARS outbreak in 2003 was still 9.33 per 100,000, which translates into an addition of approximately 1.23 million new HCV infections. At the same time, hepatitis C was one of the diseases with the fastest growing incidence, which is at 19.2% per year [31]. Fortunately, the approval of direct-acting antiviral agents (DAAs) has made HCV cure possible, and DAAs are expected to reduce HCV disease burden in China.

The present study revealed that the HCV infection rate increased as the age of the subjects became higher, and three peaks appeared at the 16–20, 41–50, and 61–65 groups.

This result was consistent with a global epidemiology study, which revealed HCV infection increased with age and peaked at 55–64 years of age group [7]. The peak at 41–50 years of age group was identified in the 2006 nationwide study, which possibly resulted from the use of unscreened blood products before the implementation of screening blood donors in the 1990s [32]. The peak at the 16–20 group and the 61–65 group was in parallel with HIV prevalence, and this was correlated to the unprotected sexual behavior among adolescents and elderly men who often visited underground prostitutes in China [33]. These two peaks suggest that sexual transmission is the major route for HCV infection [34].

HCV infection in males was significantly higher than females in the present study (*P* < 0.05). In fact, the gender difference was common in most liver diseases, as well as other disorders. It appears that the higher frequency in the male population, who are inclined to have a high-risk engagement, may make them vulnerable to HCV transmission [7].

Several factors may attribute to the difference in HCV infection between the two genders. Some studies have suggested that females tended to have higher rates of viral clearance, reclearance after reinfection, and better response to HCV treatment, when compared to the male counterparts [35–37].

It is noteworthy that some studies have suggested that some females are prone to higher HCV infection due to their professions and medical conditions, such as curettage, abortion, childbirth, or hospitalization [32, 38, 39].

The HCV infection rate varied with the geographic regions in the present study, and this was significantly higher in plains and coastal islands, when compared to hills/mountains (0.19%, 0.18%, and 0.06%, respectively, *P* < 0.001). Previous studies on HBV infection in Southeast China have also revealed that in terms of the differences among geographic regions, HBV was more common in valleys and costal islands, when compared to plains [24, 40]. The discrepancy between hills/mountains and plains could be attributable to the different transmission routes of HBV and HCV. HCV is mainly transmitted through blood contact from IVDU and unsafe medical procedures. The dense population in the plain region and its robust economy that attracts a large number of immigration labor from undeveloped regions of China have made these plain areas vulnerable to HCV exposure. In contrast, HBV MTCT (mother-to-children transmission) remains as the main transmission mode. The hill/mountain regions are mainly located in the south and west part of the Zhejiang province, where the economy is underdeveloped, the lifestyle tends to be more conservative, and the care provided by obstetrics/gynecology and pediatrics is relatively poor. All these favor a high HBV, but a low HCV transmission.

After reviewing the published data [41–44], a HCV RNA cutoff level of 10*E* + 05 IU/ml was used to separate HCV-positive samples into high and low HCV RNA groups.

High HCV RNA level is associated with advanced liver disease and may facilitate liver injury and progression [45]. However, factors that determine blood HCV RNA levels are poorly understood [46]. In the present study, both univariate and multivariate analyses identified the transfusion history as an independent risk factor for high HCV RNA levels. Similarly, a previous study indicated that the HCV RNA level was positively associated with needle sharing [47]. To some extent, this may explain the high RNA level in the transfusion group. In our study, we found out that blood transfusion is a risk factor of high HCV RNA level when compared to other transmission routes. It suggests that government is supposed to take some measures to cut off the blood transmission. As a result, there will be fewer patients with high HCV RNA level and fewer infected people with advanced liver disease [45].

The present study also revealed that patients with an urban residential history were associated with high RNA levels, when compared with the rural group. Although the mechanism remains unclear, and the investigators reckon that the frequent immigration of populations in the urban area may offer an explanation. The frequent immigrations created a greater opportunity for sharing needles among IDUs and HIV-infected subjects. In addition, previous studies have shown that both groups are associated with higher HCV viral load [47, 48].

In addition, previous studies have shown that the HCV RNA load was correlated to many factors. Ali A. et al. found that the baseline viral load was significantly higher in patients infected by genotype 3, when compared to other genotypes [49]. Although gender was not a significant risk factor for high HCV RNA levels in the present study (*P*=0.20), and some studies have indicated that females tended to have a higher rate of viral clearance [35]. Hence, more data from large-scale studies are needed to ascertain the risk factors and mechanism. HCV genotype 1 globally infected 83.4 million (46.2%) people, and 1/3 of them are in East Asia. Genotype 3 ranks as the second most common genotype, with 54.3 million (30.1%) infected people. Genotype 4 and 5 appeared more frequently in low-income countries [50].

Subtypes 1a, 1b, 2a, and 3a have been frequently detected in high-income countries, and these have been viewed as the “epidemic subtype” [51, 52]. Other subtypes appeared to be geographic specific. For instance, genotype 3 is distributed mainly in South Asia, while genotype 6 is distributed mainly in Southeast Asia [53].

Previous reports have found that HCV genotypes in China and East Asia resembled the global distribution, except for genotype 6, which was 16.2%, and was only secondary to genotype 1 (58%). Genotype 2 and 3 accounted for 15.3% and 10.4%, respectively [50].

The present genotype results were, to a large extent, consistent with the genotype distribution in China. Genotype 6 was slightly higher than the average level of 16.2%, which may have resulted from the geographic reason because Southeast China has frequent personal and commercial connections with Southeast Asia, in which genotype 6 is dominant. Genotypes 4 or 5 were not detected. This implies that the same treatment regimens used by other regions in China are applicable in Southeast China.

An obvious limitation in the present study was that this hospital-based population may not represent the community-based population. A community-based study, such as a national serologic survey, is a standard epidemiologic study, but it is expensive to conduct. An alternative is to collect data from large health care institutions, including both outpatient and inpatient populations. Prior to the 1990s, the US Veterans Affair Department conducted a national survey on serum HCV antibody status among veterans [54]. In addition, the US Cherokee Nation Health Services (CNHS) conducted a HCV infection survey among patients who were in their health system [55]. It was considered that this limitation, to some extent, was mitigated by the very large size of the sample included in this study.

## 5. Conclusion

In the present large-scale study of HCV infection epidemiology, it was found that HCV infection occurred in 0.17% of the general population in Southeast China, and this prevalence is lower than that in the 2006 national survey. In the present studied population, HCV infection was significantly higher in males than in females.

Furthermore, the HCV infection rate varied with different geographic regions, and more HCV infections were detected in plains and coastal areas than in valleys. HCV genotype 1 was found to be dominant, followed by genotype 6. Transfusion history and urban resident were risk factors for high HCV RNA levels. The present data update the latest HCV infection status in the Zhejiang province.

## Figures and Tables

**Table 1:** Demographic and clinical features of HCV-infected subjects (*n* = 68).

Variables	Size	
Age	45.80 ± 13.67	
Gender		
Male	42	
Female	26	
Infection route	+	−
IVDA	6	62
Hemodialysis	0	68
Transfusion	13	55
Coinfection	+	−
HBV	15	53
HIV	0	68
Syphilis	4	64
Residential area		
Rural	41	
Urban	27	
Genotype	Size	%
1b	34	50.00
2a	6	8.82
3a	3	4.41
3b	2	2.94
6a	15	22.06
6n	1	1.47
Missing data	7	10.29

**Table 2:** Univariate and multivariate analysis of risk factors associated with high HCV RNA level (*n* = 68).

Influence factors	OR	*P*	aOR	*P*
Age	0.96 (0.92 to 1.01)	0.09		
Gender				
Male	Ref			
Female	0.45 (0.13 to 1.54)	0.20		
Transfusion				
−	Ref			
+	5.88 (1.53 to 22.63)	0.01	11.24 (1.28 to 100)	0.03
Residential area				
Rural	Ref			
Urban	10.75 (1.31 to 90.91)	0.03	6.20 (1.41 to 27.35)	0.02

OR, odds ratio; aOR, adjusted OR.

**Table 3:** Univariate and multivariate analysis of risk factors for cirrhosis (*n* = 68).

Influence factors	OR	*P*	aOR	*P*
Demographic	1.03 (0.99 to 1.07)	0.17		
Age				
Gender male	Ref			
Female	0.46 (0.13 to 1.60)	0.22		
Transfusion	Ref			
−				
+	2.50 (0.68 to 9.17)	0.017		
Residential area	Ref			
Urban				
Rural	1.61 (0.49 to 5.31)	0.43		
HBV coinfection	Ref			
−				
+	0.77 (0.19 to 3.16)	0.72		
HCV RNA level	Ref			
Low				
High	0.63 (0.16 to 2.40)	0.50		
Lab tests				
Albumin	0.90 (0.82 to 0.99)	0.04		
ALT	1.01 (1.00 to 1.02)	0.04	1.03 (1.01 to 1.05)	0.01
AST	1.03 (1.01 to 1.05)	0.01		
TB	1.04 (0.99 to 1.08)	0.14		

ALT, alanine aminotransferase; AST, aspartate aminotransferase; TB, total bilirubin; OR, odds ratio; aOR, adjusted OR.

## Data Availability

The datasets generated and analyzed during the present study are available from the corresponding author on reasonable request.
